# A teaching concept for school experiments on radioactivity using augmented reality methods

**DOI:** 10.1093/rpd/ncad071

**Published:** 2023-05-24

**Authors:** Charlotte Schuette, Marcus Streuber, Vivien Pottgiesser, Bernhard Preim, Patrick Saalfeld, Jan-Willem Vahlbruch, Clemens Walther

**Affiliations:** Institute of Radioecology and Radiation Protection, Leibniz Universität Hannover, Hannover 30419, Germany; Department of Visualization, Otto-von-Guericke Universität Magdeburg/Institute of Simulation and Graphics, Magdeburg 39106, Germany; Institute of Radioecology and Radiation Protection, Leibniz Universität Hannover, Hannover 30419, Germany; Department of Visualization, Otto-von-Guericke Universität Magdeburg/Institute of Simulation and Graphics, Magdeburg 39106, Germany; Department of Visualization, Otto-von-Guericke Universität Magdeburg/Institute of Simulation and Graphics, Magdeburg 39106, Germany; Institute of Radioecology and Radiation Protection, Leibniz Universität Hannover, Hannover 30419, Germany; Institute of Radioecology and Radiation Protection, Leibniz Universität Hannover, Hannover 30419, Germany

## Abstract

Digital media are becoming increasingly influential in society, especially among the younger generation. Therefore, an augmented reality (AR) app was developed that simulates experiments with radioactive sources. The app runs experiments on the range and penetration power of alpha, beta and gamma radiation. It assigns virtual radiation sources, shielding materials or a detector to printed image markers, and superimposes their 3D images on the camera image. Alpha, beta and gamma radiation are clearly distinguishable by choosing different visualizations. The detector displays the measured count rates. At school, the app can be used in different ways. A concept for a teaching unit in Grade 10 was developed and tested in several classes based on a prototype of the app. The learning progress from the AR experiments was examined. Furthermore, an evaluation of the app was carried out. The most recent version of the app can be found here: https://seafile.projekt.uni-hannover.de/d/dd033aaaf5df4ec18362/

## Introduction

Digitalization plays an increasingly important role in schools. In Germany, digital media are to be regularly integrated into the classes as defined by the German Ministers of Education Conference in 2017^(1)^. The aim is to strengthen students’ media skills and increase their motivation by connecting school methods to everyday life^(1)^. Therefore, an augmented reality (AR) app was developed that simulates experiments with radioactive sources. AR means the virtual addition of information to a live image or video of the real world. The goal of the AR app is to visualize ionizing radiation and thus to support students’ understanding of radiation.

The students can use the app to investigate the distance that the particles travel in air and which materials shield the radiation. By placing image markers, the students can plan their experimental setups in reality. Subsequently, the app assigns radiation sources, shielding materials or a detector to the corresponding markers and superimposes them on the camera image.

The programming was done based on the relevant radiation transport equations, which are presented in the next section^(2–4)^. In parallel to the programming, a teaching concept was developed in spring 2021 for using the app in school classes.

A prototype of the app was tested in physics lessons at multiple German schools to evaluate the AR experiments and their learning outcome. Furthermore, student feedback on the function of the app was collected to determine if the experiments served their purpose of illustrating and supporting the theory of radiation. The feedback was also used to improve the app.

## The development of the AR app

The AR lab app was based on the development of the AR laboratory environment and a number of predefined requirements:

The AR environment is required to visualize alpha, beta and gamma radiation. Furthermore, simple experiments are to be performed. For the experiments on the penetration depth and shielding of ionizing radiation, the following components must be realized in the AR environment: a detector, various radiation sources and shielding materials. Before programming these components, properties of a real laboratory environment were rated with regard to relevance, and it was decided which of them to include and how realistic the modelling need to be. Semi-empirical models are used to describe the different components. For instance, each radiation source is assumed to emit only one type of radiation. This is of course a simplification but serves didactic reduction purposes as well as limitation of programming effort. The app has been developed in a modular way facilitating easy expansion if required.

### The detector

The app uses a Geiger–Müller (GM) counter detector that measures the counts per time interval. This counting tube was chosen because it is widely used, and the students will most likely recognize it from previous lessons. The counting tube can measure either count rate (counts per minute) or the absolute number of counts for a predefined time interval (e.g. 2 min). The measurement stops automatically after the selected time interval. For further applications, a dose ratemeter was also implemented, which gives the dose rate in micro Sieverts per hour. For simplicity, the dose rate and the count rate are considered to be proportional with a proportionality constant of C = 59703.6 counts μSv^−1^. This value has been calculated from data with real-life experiments. Moreover, a background is simulated with 1 count s^−1^. The background radiation can be measured and is automatically added to all measured values. In addition, an uncertainty of ±5% of the displayed measured values is assumed.

### The radiation sources and shielding materials

Alpha, beta and gamma radiation emitters need to be available for sources.

The alpha emitter is assumed to be a point source. To simplify the measurement, the beam fans out slightly with increasing distance, forming a cone shape. Two sources typically available in schools are provided, ^241^Am with an activity of A = 0.49 kBq and ^226^Ra with A = 60 kBq.

For each beam, the GM detector produces a constant count rate $ \overset{.}{\text N}$, with $ \overset{.}{\text N}$ = 58.8 counts s^−1^ for ^241^Am and $ \overset{.}{\text N}$ = 7200 counts s^−1^ for ^226^Ra. The count rate breaks off almost abruptly when the penetration depth of alpha radiation in air R_L_ is reached. According to Geiger, the range is proportional to the third power of the velocity^(5)^. Thus, R_L_ depends on the energy E of the alpha particles and can be estimated by the formula R_L_ = 0.32 E^3/2(2)^. For ^241^Am, the range in air is about R_L_ = 4 cm, for ^226^Ra it is about R_L_ = 3.3 cm.

Alpha radiation is shielded by basically any solid material. Classically, a sheet of paper is used^(6)^. Because of the low penetrating power of alpha radiation, the AR app is based on the assumption that any material completely shields alpha radiation. The counter in the app simply stops when a shield of any thickness is introduced between the source and GM detector.

The beta source is a ^90^Sr emitter of A = 2.87 kBq, modelled as a point source as well. The radiation angle of the source is reduced by apertures behind the sample, so that the radiation propagates conically away from the source. In contrast to the description of alpha radiation, a constant count rate $ \overset{.}{\text N}$ is not assumed for beta radiation. Instead, the dose rate $ \overset{.}{\text H}$ is considered. In this way, beta radiation and also bremsstrahlung arising in the shielding can be approximated. The dose rate $ \overset{.}{\text H}$ decreases with increasing distance from the source approximately proportional to the square of the distance. This assumption is not strictly valid, but it is sufficient for use in school. The relation is described by Equation (1). The decrease proportional to the squared distance is accounted for in the dose rate function f_β_. This function f_β_ was derived from data provided by Vogt and Vahlbruch^(3)^. To calculate the count rate, one has to include a downtime correction for the GM counter (Equation (2))^(7)^. Using the proportionality constant C, the count rate $ \overset{.}{\text N}$ can be determined from the dose rate via the Equation (1). Inserting it into Equation (2) allows to take the downtime of GM detectors into account. The downtime is assumed to be τ = 100 μs.


(1) \begin{equation*} {\overset{.}{\text H}}\left(\mathrm{r}\right)={\mathrm{f}}_{\mathrm{\beta}}\left(\mathrm{r}\right)\ \mathrm{A} \end{equation*}

(2) \begin{equation*} \overset{.}{\text N}_{\mathrm{\tau}}=\overset{.}{\text N}/\left(\overset{.}{\text N}\ \mathrm{\tau} +1\right) \end{equation*}

If shielding is used, weakening of radiation occurs in the material. Equation (3) calculates the dose rate after shielding. The equation calculates the dose rate at the distance of the shielding via Equation (1). It is then assumed that the beta radiation is exponentially weakened in the shielding. Though not absolutely correct, this approximation suffices for the intended use. The available shielding materials for beta radiation are polyethylene and aluminum with absorption coefficients d = 0.356 mm^−1^ for polyethylene and d = 1.084 mm^−1^ for aluminum. The thickness of the material is x.


(3) \begin{equation*} {\overset{.}{\text H}}_{\mathrm{shielding}}\left(\mathrm{r},\mathrm{x}\right)={\overset{.}{\text H}}\left(\mathrm{r}\right)\ \exp \left(-\mathrm{d}\ \mathrm{x}\right) \end{equation*}

Furthermore, bremsstrahlung occurs in the shielding. The dose rate of the bremsstrahlung is approximated by Equation (4). Here, the dose rate constant is Γ_x_/Z = 1.6 10^−4^ mSv m^2^h^−1^GBq^−1(3)^. The atomic numbers of the shielding materials are Z_eff_ = 13 for aluminum and Z_eff_ = 4.75 for polyethylene.


(4) \begin{equation*} {\overset{.}{\text H}}_{\mathrm{bremsstrahlung}}\left(\mathrm{r},\mathrm{x}\right)={\varGamma}_{\mathrm{x}}/\mathrm{Z}\ {\mathrm{Z}}_{\mathrm{eff}}\ \mathrm{A}/{\mathrm{r}}^2 \end{equation*}

The dose rate at distance r behind a shield of thickness x is obtained according to Equation (5).


(5) \begin{equation*} {\overset{.}{\text H}}\left(\mathrm{r},\mathrm{x}\right)={\overset{.}{\text H}}_{\mathrm{shielding}}\left(\mathrm{r},\mathrm{x}\right)+{\overset{.}{\text H}}_{\mathrm{bremsstrahlung}}\left(\mathrm{r},\mathrm{x}\right) \end{equation*}

^60^Co with an activity of A = 41.8 kBq and ^137^Cs with A = 18 MBq are used as gamma point sources. In contrast to alpha and beta radiation, gamma radiation penetrates the housing of the used sources since the penetrating power is significantly higher than with alpha and beta radiation^(6)^. Hence, not only a cone like beam is emitted by gamma sources but rather they are assumed to be a so-called point-like 4π source emitting equally in all directions. The dose rate decreases approximately proportional to the square of the distance. By Equation (6), the dose rate without shielding $ \overset{.}{\text H}$_0_ is calculated as a function of the distance r between the source and detector^(4)^. The dose rate constants Γ_γ_ for ^60^Co and ^137^Cs are Γ_γCo_ = 0.3544 mSv m^2^ h^−1^ GBq^−1^ and Γ_γCs_ = 0.0927 mSv m^2^h^−1^GBq^−1^, respectively.


(6) \begin{equation*} {\overset{.}{\text H}}_0\left(\mathrm{r},\mathrm{x}\right)={\varGamma}_{\mathrm{\gamma}}\ \mathrm{A}/{\mathrm{r}}^2 \end{equation*}

Behind a shielding, the dose rate $ \overset{.}{\text H}$ follows Equation (7). The reciprocal weakening coefficient for broad beam geometry 1/S(x) is a function of the layer thickness x (graphical data in Vogt and Schultz^(4)^). The primary beam is not collimated and hence the scattered radiation generated in the shielding material is considered in the broad beam geometry at the detector. The reciprocal weakening coefficient 1/S(x) depends on the shielding material and the layer thickness x^(4)^.


(7) \begin{equation*} \left(\mathrm{r},\mathrm{x}\right)=1/\mathrm{S}\left(\mathrm{x}\right){\overset{.}{\text H}}_0\left(\mathrm{r},\mathrm{x}\right) \end{equation*}

### Radiation visualization

**Figure 1:**
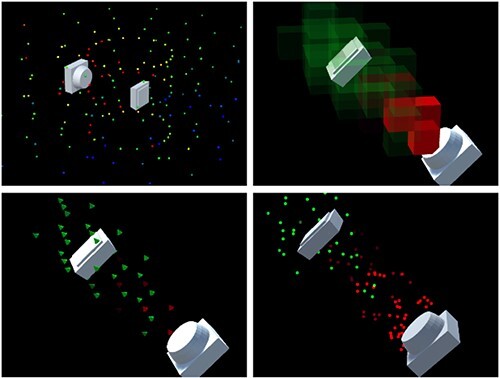
X-radiation visualization from Klunder^(8)^.

Klunder^(8)^ developed X-radiation visualizations for use with a HoloLens. In her work, she tested four visualization methods. The first represents the radiation as circles of particles, where the circle size and color visualize the radiation intensity, as can be seen in the top left of Figure 1. In the same image, the picture to the top right depicts their second visualization. Here, the world is divided into a regular grid, and the color and transparency of the chunks indicate the radiation intensity within them. The third visualization to the bottom left uses cones to not only depict the direction of the radiation flow by the cone orientation, but also the intensity at the point of the cone by its color. Lastly, the final visualization to the bottom right of Figure 1 animates spheres flying away from the radiation source and changing color depending on the intensity. Thus, it shows the radiation flow by movement. In her tests, she concluded that the last visualization with the moving particles has performed by far the best.

**Figure 2:**
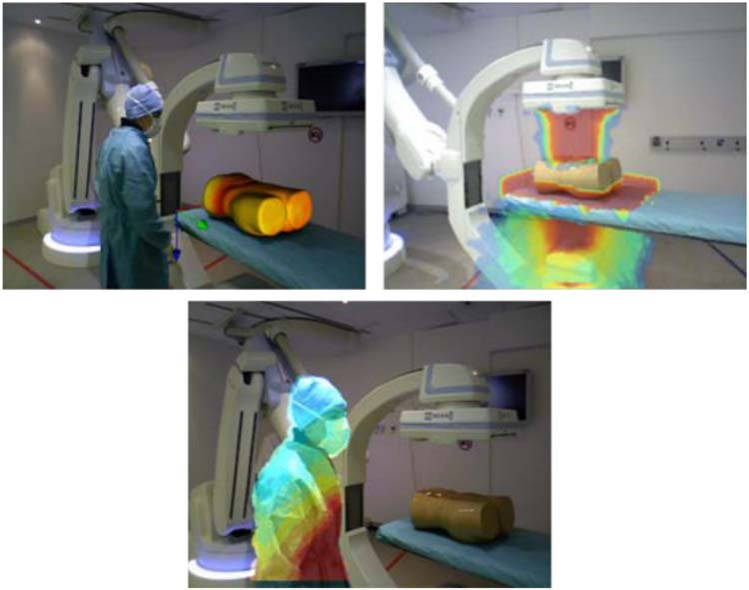
Radiation overlay by Rodas *et al*. divided a virtual room in three different modes. At the top left is the distribution on the patient, at the top right is the distribution in the room and at the bottom is the distribution on the staff. Images taken from Rodas *et al*.^(9)^.

An approach by Rodas *et al*. displayed the radiation intensity as a transparent color overlay in AR^(9)^. Rodas *et al*. split the AR radiation visualization into three different modes, as can be seen in Figure 2. Each mode uses the same color palette from blue to red to display the radiation intensity, while overlaying the visualization on a different object. The first mode shows the radiation distribution as it arrives at the patient, as can be seen in Figure 2 at the top left, whereas the second mode shows radiation in the entire room. The last mode displays the radiation as it arrives at the staff, as can be seen in Figure 2 at the bottom.

**Figure 3:**
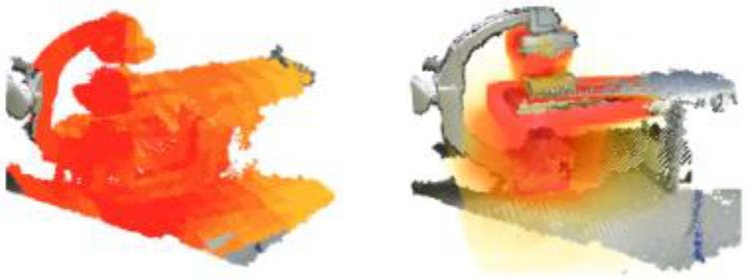
Radiation overlay by Rodas *et al*. showing the radiation at the voxel of the room (left) and voxel of the scattered radiation volume (right). Images taken from Rodas and Padoy^(10)^, reproduced with permission from SNCSC.

A similar but non-AR approach by Rodas *et al*. divided a virtual room into voxels and calculated the radiation for every voxel in the room as a risk map^(10, 11)^. By overlaying this risk map at the point cloud of the room, the radiation intensity at all surfaces can be shown, as seen on the left in Figure 3. By also using voxels that lie in the air, radiation volumes like the scatter volume in the room can be displayed transparently. Latter can be seen on the right side in Figure 3.

### The programming and visualization

The AR App for Android and iOS was developed using the Unity Engine v2020.3 and the AR Foundation. The user is able to place a source, a shielding material and a detector using image markers in the real world. These image markers are then tracked using ARCore’s (Android) and ARKit’s (iOS) image tracking. For the visualization of radiation, multiple methods were developed to be later evaluated in the user study at German schools. In the application, the user can switch between four visualization modes. These are:

(1) Particle visualization
(2) Flow visualization
(3) Both previous visualizations combined
(4) No visualization

Each method was designed to apply for each of the radiation types. The implementation of the flow visualization was done using Shader Graph in Unity which requires a custom render pipeline. For this, the Universal Render Pipeline was used. These aforementioned Shader Graph tool allows for a visual composition of a shader using a web of notes. For the particle visualization, a particle system was used. Here, particles are shot in one direction at a given rate with a given lifetime.

The visualization of radiation must clearly distinguish optically between alpha and beta radiation as particle radiation and gamma radiation as electromagnetic radiation.

#### Particle visualization

The first method mimics radiation behavior by using a particle system, where particles are emitted by the source. It is assumed that the higher the radiation intensity, the more particles are spawned, and depending on how fast intensity decreases over distance, the particle’s lifetime was chosen. For alpha and beta radiation, the appearance of these particles was selected to show the physical composition.

**Figure 4:**
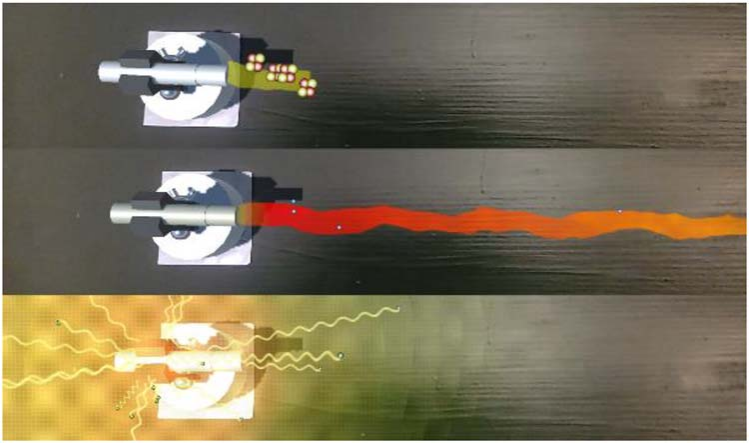
Implementation of the visualization of alpha (top), beta (middle) and gamma (bottom) radiation and their propagation in the AR app.

An alpha particle consists of two protons and two neutrons. They are represented by spheres of different color, clustered to an entity of four spheres. This can be found in Figure 4 at the top.

Beta particles, i.e. electrons, are visualized by smaller single spheres, as can be seen in Figure 4 in the middle. Alpha and beta particles are emitted into a single direction, since it is assumed that the radiation is reduced by apertures behind the sample. Within the cone-shaped beam, the particles are moving away from the source.

The gamma source is assumed to emit omnidirectionally. The particles here were emitted from the source in a spherical shape with each particle having a random direction. Since gamma radiation is electromagnetic radiation, the wave-particle duality was used to visualize the ‘particles’, i.e. the photons. This can be seen in Figure 4 on the bottom.

#### Flow visualization

The second visualization is depicting the radiation flow. The flow of alpha and beta radiation is drawn as an animated line. This can be seen in Figure 4, where the color of the line represents the intensity. An animation causes a curved line to look like it is moving, by changing the position and distortions on the line over time, and thus showing the flow direction of the radiation. For the distortion, the Simple Noise of Shader Graph is used.

In addition, this line is colored to represent the intensity of the radiation at a given point. This means that for the near-constant alpha radiation, the line visualization only uses a single color, as can be seen in Figure 4 at the top. The line for beta radiation changes color over distance, because it is considered that the energy released by beta decay is distributed arbitrarily to a neutrino and an electron. Hence, beta radiation has a continuous energy distribution with only an upper limit. Since electrons penetrate deeper into matter (or air) with increasing energy, there is no sharp cutoff at a certain depth but rather a fading, with only the most highly energetic electrons making it to the tail of the cone.

For the interaction with shielding materials, the line visualization gets cut of at the point where the line hits the material. The resulting transmission is a new line visualization that starts behind the shielding material and has a reduced length and color selection, depending on the used material and its width.

For the gamma radiation, a different solution for the visualization was developed. Gamma radiation, being electromagnetic radiation, is visualized as visible light. Here instead of a ray going in one direction, intensity clouds were put around the radiation source to depict the omni-directional spread of the gamma radiation. The clouds are, like the rays, animated using noise over time, giving the illusion of a fuzzy cloud instead of a well-defined sphere. The visualization uses multiple transparent clouds stacked within each other to create an increasing color intensity towards the center. To avoid this creating an effect of clear intensity borders at the rim of each cloud, the spheres are fading out towards their end. This can be seen in Figure 4 at the bottom.

**Figure 5:**
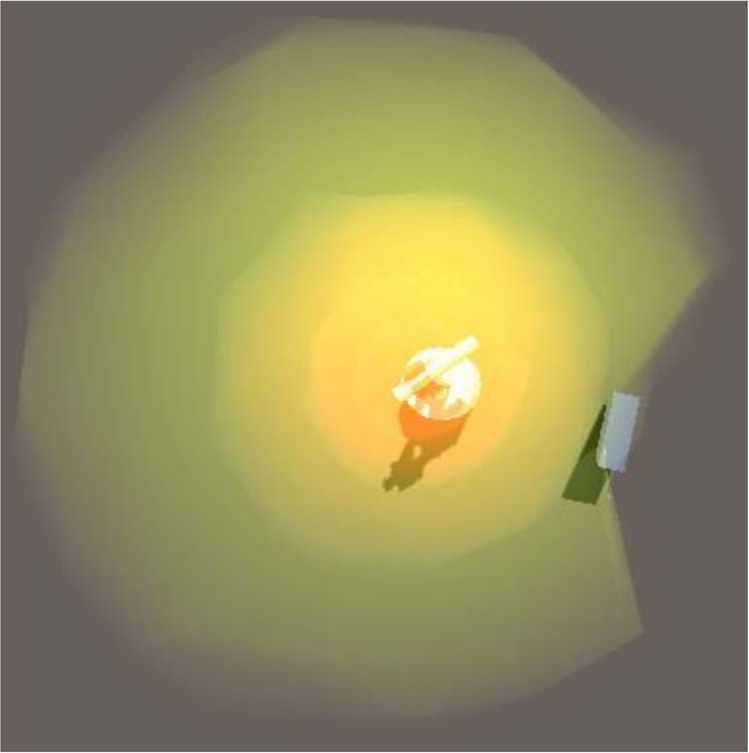
The flow visualization of gamma rays was realized with the help of intensity clouds, a small object at the bottom right shields part of the radiation, resulting in a dent in the cloud.

The interaction of the gamma radiation with shielding materials can be seen in Figure 5. A small object is placed at the bottom right and shields part of the radiation, resulting in a dent in the cloud visualizing gamma radiation. To better display the shielding behavior, the dithering has been deactivated in this image.

### The function of the AR app

The AR app can be downloaded on Android and iOS devices. To carry out the experiments, the so-called image markers (QR codes) are needed. The image markers are recognized by the app and the respective experimental devices can be superimposed. The most current version of the app and the image markers are publicly available here: https://seafile.projekt.uni-hannover.de/d/dd033aaaf5df4ec18362/

After starting the App, the logo of Euratom, European Atomic Energy Community appears to acknowledge the financial support. Touching the display makes the logo disappear. By using the camera, the smart device images the scene in front of the user.

The visualization can be customized by the user:

(1) At the top right-hand corner, three dots lead to a selection menu. The menu allows four different visualization settings: either the particles like the helium nuclei, in the case of alpha radiation, are displayed on their way from the source to the detector or only the cone will be visible indicating the propagation of the radiation. Alternatively, both can be displayed together or even neither.
(2) The names of the radiation sources can be turned on or off.
(3) In the newer versions of the app, the graphics resolution can be adjusted.
(4) On the main screen in the top left-hand corner, one can select German, English, French, Spanish or Russian language.
(5) In the lower right-hand corner, a dose rate is displayed. The value corresponds to the dose rate, which a real detector would detect at the position of the smart device in the student’s hand.

Upon scanning of one of the image markers, a corresponding AR device appears on the screen that is either a detector, a shield or a radiation source. At the time of the school visits, it was possible to choose between the four different sources: ^226^Ra, ^241^Am, ^90^Sr, ^60^Co. In the meantime, ^137^Cs has been added for additional experiments.

After selecting a radiation source, its name, the type of emitted radiation and the activity of the source are displayed. Only numbers and activities are shown in case the name display is switched off (Option 2).

Either a GM counter or a dose rate meter can be chosen for detection of the radiation. The GM counter displays values in counts s^−1^ or counts per reselectable time unit. The dose rate is given in nSv h^−1^.

Five different materials can be selected for a shielding material: polyethylene, lead, concrete, iron and aluminum. The thickness of each material is adjusted by the use of a slider.

To setup the AR environment, the user scans the markers individually and from a short distance. Performing experiments with the app includes moving or rotating the measuring devices. It is important to re-scan the markers after they have been moved, as the app remembers the position of the markers otherwise. In case devices are moved unintentionally, the scanning process can be restarted by briefly covering the camera.

### The experiments

Currently, the app supports the following experiments. The background is obtained by starting the measurements without having scanned the image marker of a source before.

The distance that the alpha particles travel in air can be determined. Moreover, it can be investigated which materials shield the radiation. Any of the available shielding materials (Polyethylene, lead, concrete, iron and aluminum) can be placed between the source and the detector. By the same principle, students can study beta and gamma emitters. In case of the beta emitter, polyethylene and aluminum of various thicknesses can be used for shielding. The penetrating power of gamma radiation is measured by shielding with lead, iron or concrete.

**Figure 6:**
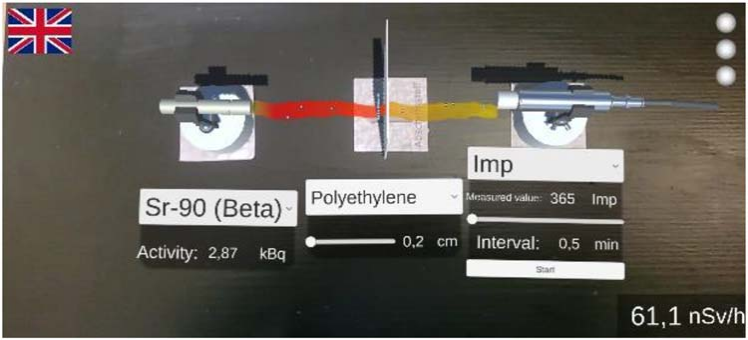
An exemplary setup in the AR app. ^90^Sr was chosen as a source and polyethylene as a shield. The count rate for a time interval of 30 s can be found on the detector.

An exemplary set-up of one of the experiments can be found in Figure 6. ^90^Sr was chosen as a source and polyethylene as a shield. The count rate for a time interval of 30 s can be found on the detector.

## The teaching concept

At school, the app is used in a variety of ways. In sequential lessons, the students can work out the characteristics of ionizing radiation. Alternatively, the app can serve for repeating and consolidating knowledge obtained in previous units on radioactivity. For both options, students are actively engaged in familiarizing themselves with the features of the app. Subsequently, they conduct various AR experiments. Students are allowed to discover the important functions of the app and work independently following the approach of enquiry- and discovery-based learning as described by Bell^(12)^.

However, some basic knowledge of the students on radioactivity is required to smoothly run the AR experiments. The topics that will be relevant for the lessons with the AR app rely on this.

### Prior knowledge of the students

Recommended basic nuclear physics knowledge includes:

- A nuclide is an atom, which is characterized by the number of protons Z and neutrons N in the nucleus, and the definition of the atomic mass is A = Z + N.
- The term isotope denominates a nuclide with equal atomic number Z and different neutron numbers N.
- The chart of the nuclides orders isotopes according to the proton number Z and neutron number N.
- The nature of alpha, beta and gamma radiation. This is important to understand the visualization of radiation in the AR environment.

These basic topics should be revisited at the beginning of the lesson due to the great relevance for understanding the visualization.

To complete the lesson, detailed knowledge of how a GM counter works is not required. It might be briefly explained during the course, however, that there are devices for detecting ionizing radiation other than the GM counter along with their preferred applications.

Very important is the understanding of the measured quantities: the count rate, e.g. counts per second, indicates how many radiation particles are registered in the detector per time. This should not be confused with the activity of the source, given as nuclear decays per second in Becquerel. Usually, the efficiency of the detector is much lower than one, i.e. the activity of the source far exceeds the measured count rate. It goes beyond the scope of the present lesson, however, to perform an efficiency calibration. Last but not the least, the background radiation should be addressed as a consequence of natural radioactivity.

### The concept

Based on the aforementioned approach of enquiry-based learning, the *first phase* of working with the app should consist of an orientation activity. The students explore the AR environment and discover the function of the image markers. In the following discussion, all the relevant functions of the app are named and the visualization is explained. This is done by repeating the nature of the different types of radiation. This discussion relates theoretical knowledge to the practical application and lays the foundation for the experimentation phase.

In the *second phase*, an overall research goal is determined. Depending on the class and the lesson topic, this is either done independently by the students or guided by the teacher. The research goal puts the experiment into perspective and provides a central theme for the lesson, which can be revisited at its end. A possible goal would be, ‘We will investigate the characteristics of ionizing radiation in the AR environment and make statements about range and penetrating power.’ This goal is very general and can be further narrowed down for specific lessons, for instance on either of the three kinds of radiation or specific shielding materials.

The *third phase* consists of planning the enquiry, i.e. execution of the experiments. Experimental protocols can be created as a structuring aid. The teacher should support this step. For classes that have little experience in planning experiments independently, experimental protocols should be provided for guidance. There is a certain risk that the transfer from the experimental setup in reality to the image markers and the experimental execution in the AR environment causes problems. To simplify this step, the experimental setup in AR versus reality should be discussed in detail. This discussion of the setup is recommended for all classes.

In the *fourth phase*, the students’ expectations on the behavior of ionizing radiation are subsumed to one or more scientific hypotheses. These hypotheses are subsequently tested with the planned experiments. To this end, the students perform measurements with the AR app as described above and evaluate the data. The evaluation can be adapted to the knowledge of the class and the respective lesson goals. In the trial lessons carried out in multiple German schools, the measured values were displayed graphically and described by equalization functions. If the measurement results are plotted as a graph, the trend can be approximated. For example, if the count rate of beta radiation is measured at different distances from the source, the count rate could be plotted against the distance. This would result in an approximately exponential curve. If the experiment is carried out correctly, a similar tendency of the measured values should emerge for all students. This eases discussion in plenary in the next phase without using the multitude of measured values directly. At the same time, this reduction circumvents the problem of slightly different values the students take due to different placement of the markers and to the programmed small random component of the generated data. These tendencies are discussed as qualitative statements in the plenary. In the *last phase*, the results should be adequately documented.

These steps provide a general structure for working with the app. Nevertheless, the students also have the opportunity to contribute their own ideas.

### The implementation in class

The step-based method can be adapted for different purposes.

When investigating the characteristics of ionizing radiation, the method presented can be repeated twice. First, experiments on the range (in air) of alpha, beta and gamma radiation can be carried out. Second, after a detailed discussion of the ranges in air, a lesson on penetrating power and shielding of different materials is conducted. In this second lesson, the orientation phase can be omitted since the students are already familiar with the app.

If the entire unit on radioactivity is to be repeated using the app, the methods can also be adapted. The app offers the possibility to switch off the names of the radiation sources and the visualizations. If the names and the visualizations are switched off, the experiments on range and penetrating power can be carried out ‘blindly’. Students record the measured values for all sources and compare with the known properties of the radiation types. In the end, the visualizations can be switched on for a verification check.

These two variants were used for the trial lessons in German schools to test the app and the teaching concept.

Of course, there are other ways to use the app in the classes. For alpha and gamma sources, for example, it is now also possible to compare two sources of the same type of radiation. Alternatively, the app can be used alongside classical lessons or for the preparation of more advanced hands-on experiments. Furthermore, the app can be used repeatedly for illustration, such as the distance-squared law. Regardless of how the app is to be used in class, the teacher must take some time in advance to get to know the app and its functions to answer students’ questions. For a quick and successful start in working with the AR app, a short tutorial of one page is available together with the download of the AR app.

## Evaluation of the student surveys

Four German school classes worked with the app for testing the teaching concept. On the one hand, the learning progress of the students was tested after performing the experiments with the AR environment. On the other hand, the students were requested to provide feedback on the functions of the AR app and the visualizations.

The implementation of the lessons went well in all classes. The method was well received by the students. In the orientation phases, the students were able to find and try out all the important functions on their own.

When carrying out the experiments, some assistance was necessary regarding the handling of the app. Nevertheless, at the end of the experimentation phase, most of the students had obtained eligible results allowing further evaluation.

In general, the evaluation phases turned out to be very short, as time was very tight. For regular use in class, sufficient time should be planned for evaluation and discussion of the results.

The students gained important experience in handling the app during the lessons. They provided valuable feedback for implementation of further improvements.

To measure learning progress, the students were asked the same questions about ionizing radiation and its characteristics before and after the lesson. A comparison of both tests revealed positive results, showing that over 50% of the students showed learning gains due to working with the app. The visualization contributed to a better understanding of the composition and propagation of ionizing radiation. A detailed discussion of the given radiation sources in comparison to other emitters proved to be important. For example, the measured range of radiation from the ^90^Sr source is not easily transferable to all beta emitters due to the energy dependence and the different maximum energies of the spectra.

The fourth class’s learning progress turned out to be quite diverse. Due to short-term Covid-19 restrictions, the first lesson was taught with only half of the class and the next lesson with the whole class. Hence, the students had different tasks in the second lesson, which complicated the evaluation and led to increased unrest in the group.

The feedback from the 99 students aged 14–17 interviewed showed that 77% considered the experiments in the AR app to be a diversifying alternative to normal physics lessons. The new teacher who came especially for the experiments in AR might also have had an influence on these results. Nevertheless, 55% of the students enjoyed experimenting with the app. Sixty-nine per cent of the students agreed that the app was easy to use. The students also liked the idea of the app, and the visualization was particularly well received. One student wrote: ‘You understand how it works with the radiation, and how and where it spreads.’

In evaluating the visualization, where the four methods (flow, particle visualization, both, none) could be assessed individually, participants were tasked to grade each visualization to their educational usefulness as well as their visual appeal. The use of both visualizations is graded slightly better than flow-only and the use of only particles slightly worse than the flow-only visualization. To test for statistical significance, one-way analysis of variance was conducted between the pairs of visual appeal ratings as well as pairs of usefulness ratings. It was found that there were no statistically relevant differences in the visualization evaluation. Finally, only 35% of the students felt the experiments in AR cannot replace real experiments.

On the technical side, students provided constructive suggestions for improvements. For example, aligning the devices was very difficult because of the short penetration depth and restricted measurement geometry of alpha radiation. In the updated version of the app, the radius in which the measured value can be recorded by the detector was enlarged.

In addition, the app did not always run smoothly at times or completely stalled. To safe computing power, the updated version of the app includes the option to reduce the display resolution. This enables the app to run smoothly on most devices.

## Discussion

The question whether the AR app is better than real experiments cannot be answered easily. If possible, experiments should take place in reality. The students interact directly with the equipment, which often is a great advantage. However, not all schools can offer hands-on experiments due to the lack of radioactive sources and detectors. In this case, the use of the AR app is a valuable alternative. By visualizing the radiation, the app can also provide insights into the nature of ionizing radiation, which is not possible by hands-on experiments. For this very reason, the app is also a good supplement to real experiments.

The app definitely requires less experiment preparation effort. Once the app is installed on a number of devices and the image markers are printed, the AR experiments can be repeatedly used without further preparation.

In addition, the difference between real experiments and experiments in AR provides a good basis for discussion. At the end of the unit, a reflection on the results should take place in class. By using the AR environment, a possible topic for reflection would be why the experiments are not conducted as real experiments.

More often than not, lack of radioactive sources is the reason. However, this can be deepened by discussing costs but also potential risks of handling radioactive sources.

Another reason for choosing the AR app might be that visualizing the radiation supports understanding. In this case, a discussion about the advantages and disadvantages of virtual experiments could follow in the spirit of digitalization.

A further topic could be the strict safety rules for handling radioactive sources.

The AR app offers the possibility to use different detectors. In addition, the smart device itself can be used as a simulated detector. The general radiation readings at the bottom of the display at some distance from the experiment can be compared with the values displayed by the detector directly behind the source. This could help to initiate a discussion on said safety rules.

In addition to the experiments, the app offers further possibilities for the design of the lesson.

## Conclusion

In conclusion, the app was already working very well at the time of the class visits. Radiation was simulated well based on the equations discussed above. The data from the first series of measurements were promising. Furthermore, the representations of the devices were close to reality and the visualizations of the alpha, beta and gamma radiation were appealing. Although the prototype still showed a few weaknesses, it was already possible to experiment very well with the app. Since then, the app has been improved based on the suggestions from this research project.

The implementation of the lesson planning in the classes mostly worked smoothly. The majority of the students enjoyed working with the app and were able to take away some insights from the experiments. The evaluation showed that more than half of the students had made learning gains by the end of the lesson. The students’ evaluations of the app were also largely positive.

The execution in the school showed that the app and the implementation in the classroom needed to be revised in some places. The workload of carrying out all the experiments in a 90-min lesson was feasible, but not ideal. With more time for the individual experiments and their evaluation, the learning gain can probably be substantially increased.

In summary, the app is an enrichment of the lessons and a valuable use of new media. The evaluation confirms that this was also perceived as such by most of the students. Once the app has left the prototype stage, it can not only be used to methodically enrich physics lessons, but it can also provide students with AR-supported learning growth in terms of knowledge about ionizing radiation.

## Funding

The project leading to this application has received funding from the Euratom Research and Training Program 2014–2018 under grant agreement No 945301.
